# Supplementary material for: Platelets Inhibit Methicillin-Resistant Staphylococcus aureus by Inducing Hydroxyl Radical-Mediated Apoptosis-Like Cell Death
Source: Microbiol Spectr. 2022 Jul 19;10(4):e02441-21. doi: 10.1128/spectrum.02441-21 (PMC9431477; doi:10.1128/spectrum.02441-21)
Supplement: Supplemental file 1 — Supplemental material. Download spectrum.02441-21-s0001.pdf, PDF file, 0.9 MB [file spectrum.02441-21-s0001.pdf]

Supplemental materials

Figure S1

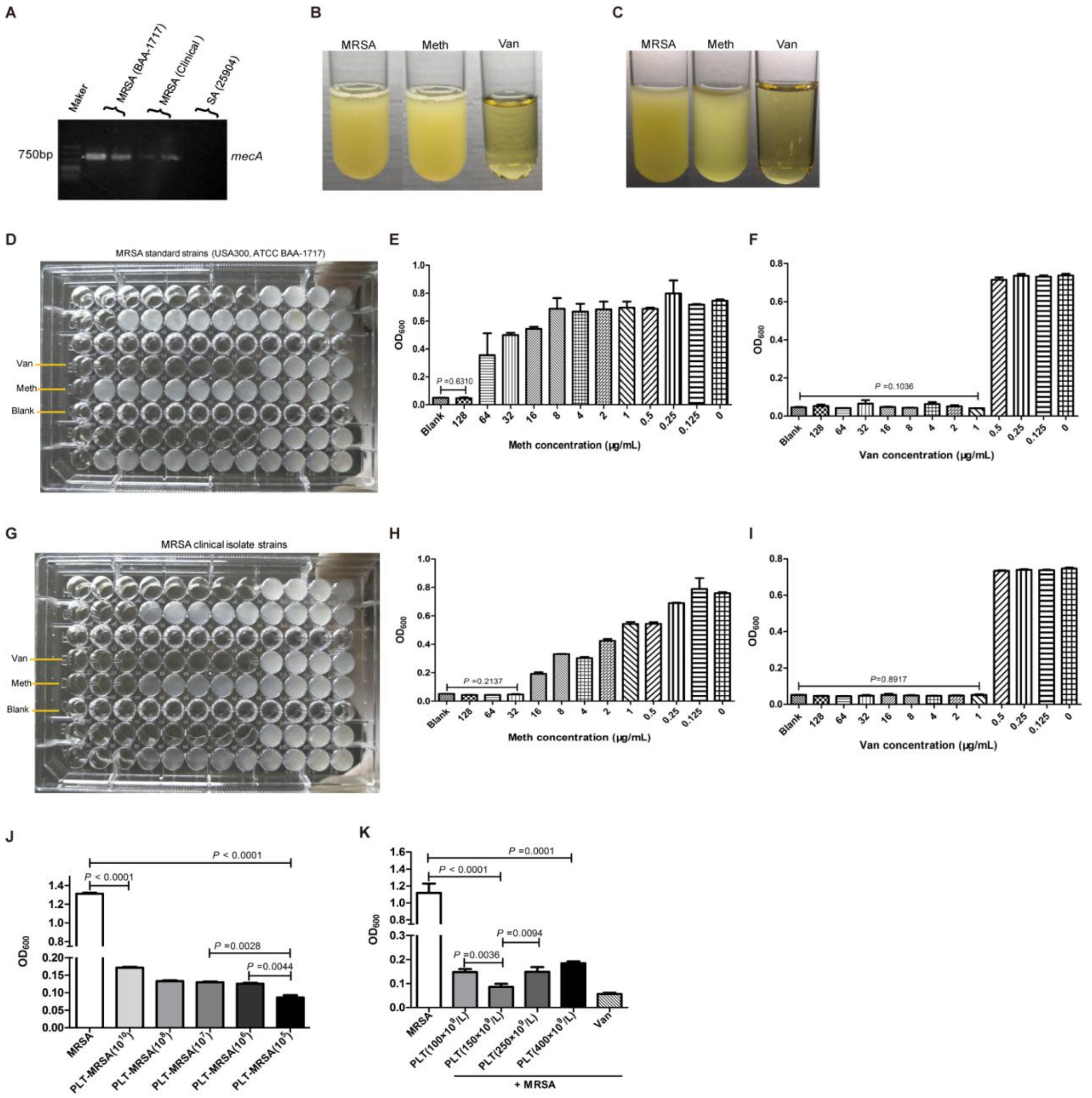

Figure S1 Identification of MRSA resistance gene *mecA* and phenotypic resistance, and establishment of the optimal system for platelet inhibition of MRSA growth. (A) Results of agarose gel nucleic acid electrophoresis identification after amplification of MRSA *mecA* gene. MRSA(BAA-1717): standard MRSA strains (USA300, ATCC BAA-1717); MRSA(clinical): clinical isolate MRSA strains; SA(25904): *Staphylococcus aureus* (ATCC 25904). (B) The photos of turbidity of standard MRSA strains treated with Meth and Van. (C) The photos of turbidity of clinical isolated MRSA strains treated with Meth and Van. MRSA: MRSA alone; Meth:

MRSA treated with methicillin (6.83 mg/L); Van: MRSA treated with vancomycin (4 mg/L). (D) The photos of MICs of Meth and Van for MRSA standard strains using a 96 well plate. (E) The OD_{600} of MIC of Meth for MRSA standard strains. (F) The OD_{600} of MIC of Van for MRSA standard strains. (G) The photos of MICs of Meth and Van for MRSA clinical isolated strains using a 96 well plate. (H) The OD_{600} of MIC of Meth for MRSA clinical isolated strains. (I) The OD_{600} of MIC of Van for MRSA clinical isolated strains. (J) The platelets inhibition of MRSA growth on different concentration of bacterial. (K) Detection of different concentration of platelets inhibiting MRSA growth. All results have been tested at least three times. Data presented as mean ± SEM. Student's *t* test for 2-group comparisons.

Figure S2

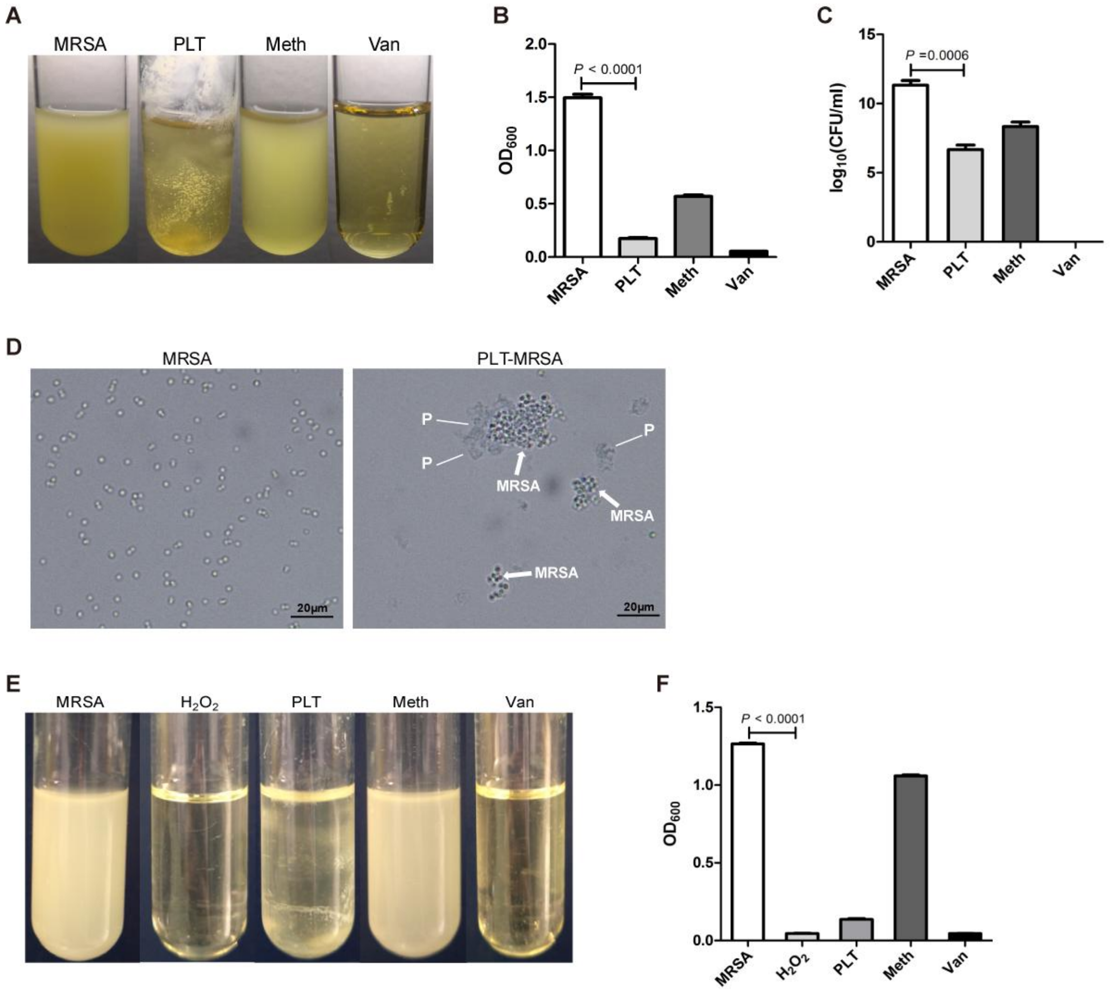

Figure S2 Platelets inhibit the growth of clinical isolated MRSA strains *in vitro*, the photos of platelets inhibiting MRSA using oil microscope with trypan blue and H_2O_2 inhibit the growth of MRSA *in vitro*. (A) The photos of turbidity of clinical isolated MRSA strains co-cultured with or without platelets for 10 hours. (B) The OD_{600} detection of each group in (A). (C) The bacterial colony counts of each group in (A). MRSA: MRSA alone; PLT: MRSA treated with platelets; Meth: MRSA treated with methicillin (6.83 mg/L), as a negative control; Van: MRSA treated with vancomycin (4 mg/L), as a positive control. (D) MRSA and MRSA co-cultured with platelets for 10h were stained with Trypan blue and observed by oil microscope. P: platelets. The original magnification was 1000×. Representative images of three independent experiments. (E) The photos of turbidity of MRSA treated with or without H_2O_2 (10 mM) for 10 hours. The other groups served as controls. (F) The OD_{600} detection of each group in (E). All results have been tested at least three times. Data presented as mean ± SEM. Student's *t* test for 2-group comparisons.

Figure S3

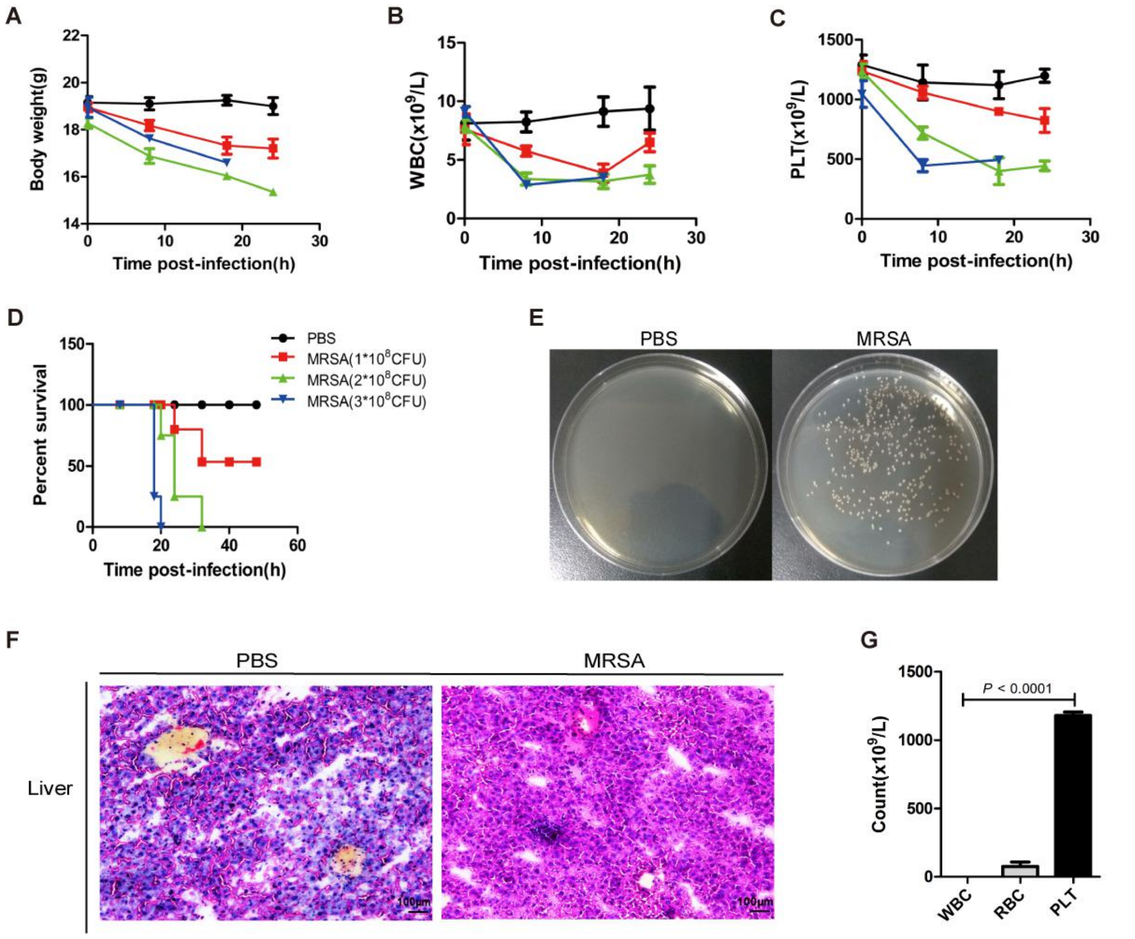

Figure S3 The mice model of MRSA infection. 1×10^8CFU, 2×10^8CFU, 3×10^8CFU MRSA were injected into mice through tail vein (n = 5 mice per group). Physiological indexes of mice in each group were detected (A-D). (A) Changes in body weight. (B) Counts of WBCs. (C) Counts of platelet by blood routine examination. (D) Monitoring of survival time-dependently. (E) Bacterial colony plate counts at 24 h. (F) The photos of H&E stain analysis of liver. The original magnification was 200×. (G) The concentration of platelets isolated from whole blood of mice was determined. Data presented as mean ± SEM. Student's *t* test for 2-group comparisons.

Figure S4

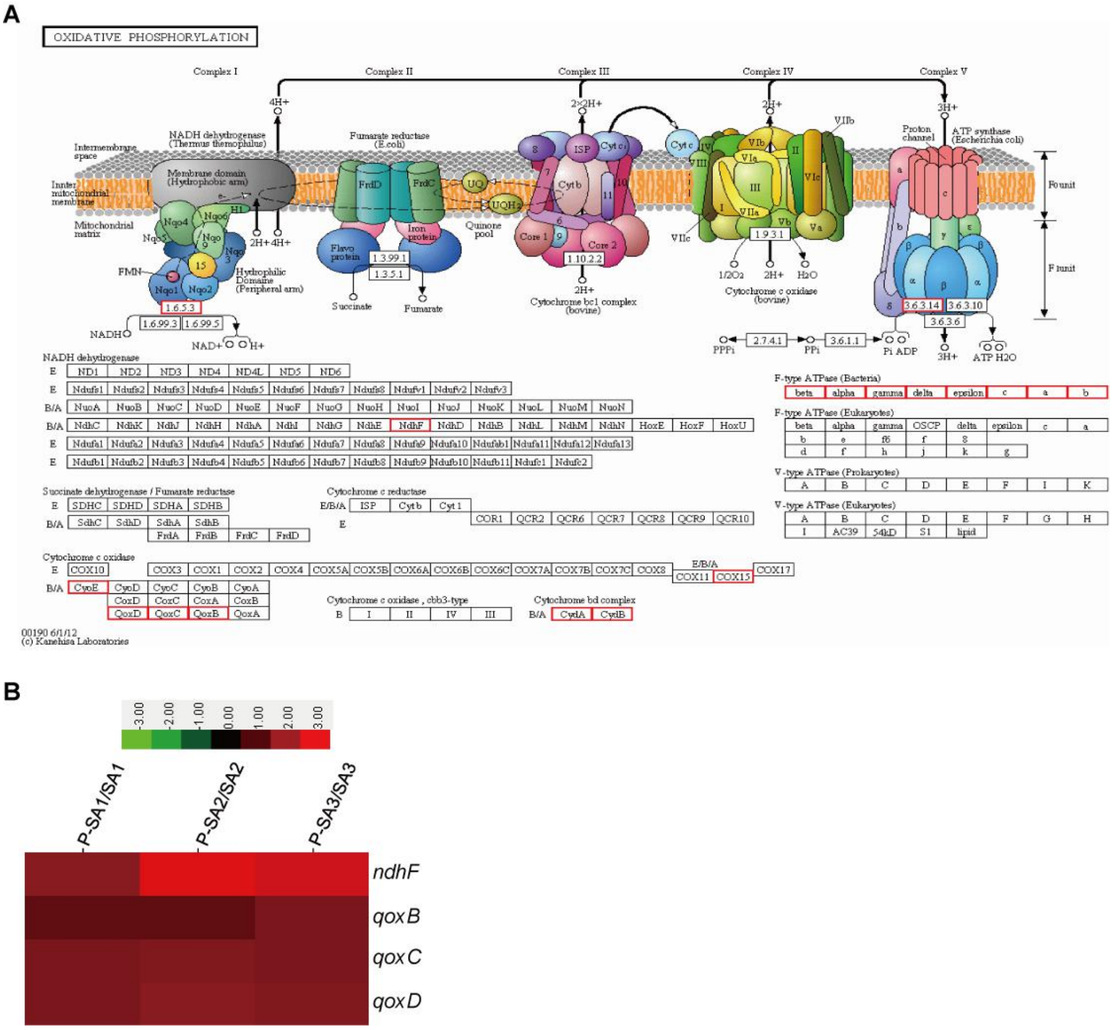

Figure S4 Three samples of *S. aureus* co-cultured with platelets for RNA-Seq. The data of RNA-Seq from three samples were compared with database. (A) Diagram of oxidative phosphorylation signaling pathway including the differentially expressed genes from RNA-Seq. The up-regulated genes are shown with red boxes. (B) Clustering analysis of *S. aureus* oxidative respiratory chain related genes in RNA-sequence results. Red represents up-regulated of genes. SA: *S. aureus,* P-SA: platelet treated *S. aureus*. P-SA1/SA1, P-SA2/SA2, and P-SA3/SA3: respectively represent the gene changes of three groups of platelet treated *S. aureus* compared with *S. aureus*.

Figure S5

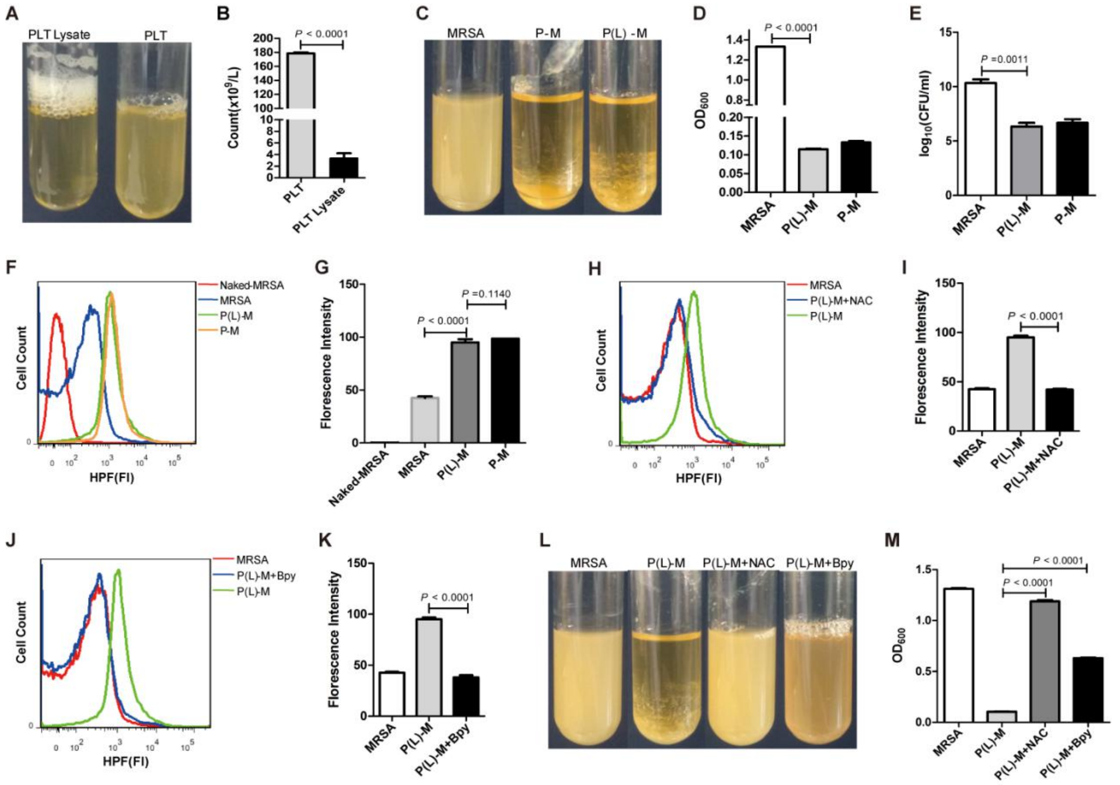

Figure S5 Platelet lysates could inhibit the growth of MRSA, in which OH• play a key role. MRSA were co-cultured with or without platelet lysates for 10 h, adding 6 mM NAC and 500 μM Bpy respectively to the coculture system. Naked-MRSA: unstained MRSA; MRSA: untreated MRSA, as a control; P(L)-M: platelet lysates treated MRSA; P-M: platelets treated MRSA, as a positive control. P(L)-M+NAC: NAC was added to the system of platelet lysates and MRSA co-culture. P(L)-M+Bpy: Bpy is added to the system of platelet lysates and MRSA co-culture. (A) Comparison of turbidity between platelet lysate and platelet suspension. (B) The numbers of platelet in platelet lysates was counted by blood routine examination. (C) Comparison of turbidity of bacterial suspensions in each group. (D) OD_{600} detection of each group in (C). (E) Bacterial counts of each group in (C). (F) The formation of OH• detected by flow cytometry. (G) Statistical results of FITC-HPF fluorescence intensity in (F).

(H) The formation of OH• detection after adding NAC. (I) Statistical results of fluorescence intensity in (H). (J) The formation of OH• detection after adding Bpy . (K) Statistical results of fluorescence intensity in (J). (L) Comparison of turbidity of bacterial suspensions in each group after adding NAC or Bpy. (M) OD_{600} detection of each group in (L). All results have been tested at least three times. Data presented as mean ± SEM. Student's *t* test for 2-group comparisons.

Figure S6

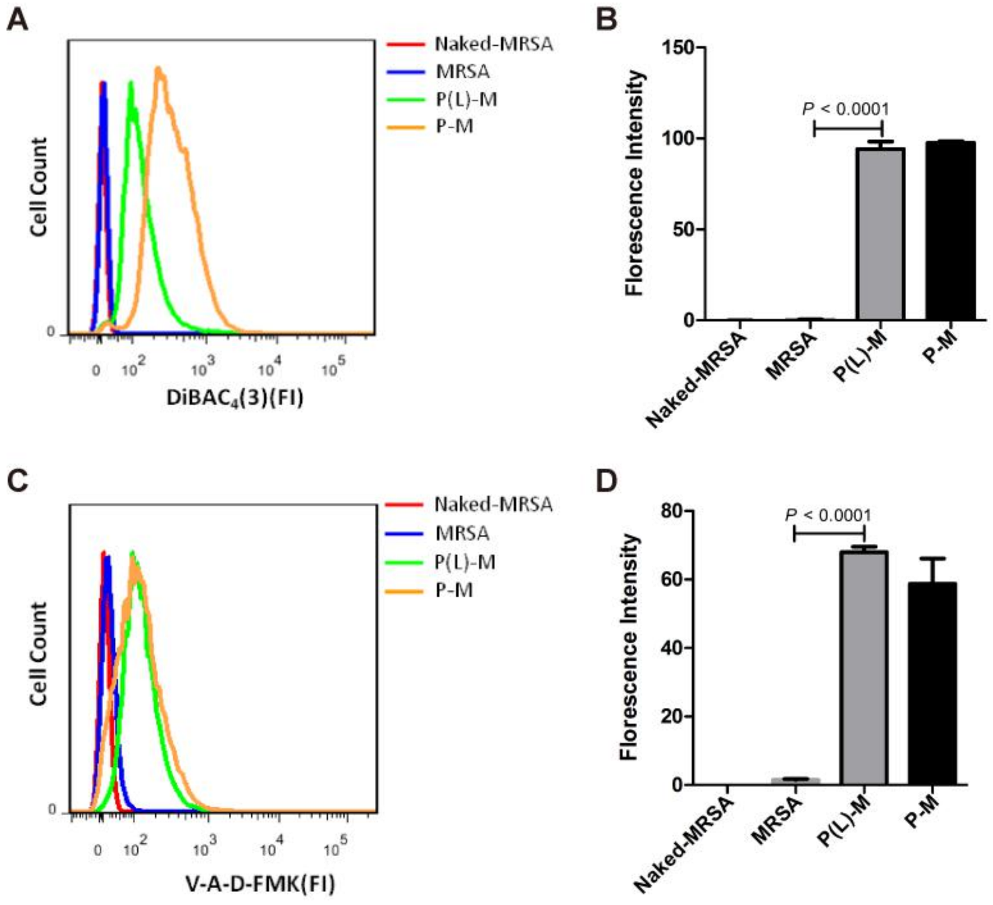

Figure S6 MRSA exhibit apoptotic characteristics after platelet lysate treatment. MRSA were co-cultured with or without platelet lysates for 10 h. Naked-MRSA: unstained MRSA, as a negative control; MRSA: untreated MRSA; P(L)-M: MRSA treated with platelet lysates; P-M: MRSA treated with platelets, as a positive control.

(A) Detection of DiBAC$_4$(3) labeled bacterial membrane potential by flow cytometry. (B) Statistical results of FITC-DiBAC$_4$(3) fluorescence intensity in (A). (C) Detection of V-A-D-FAM labeled intracellular caspase of bacteria by FACS analysis. (D) Statistical results of FITC-V-A-D-FAM fluorescence intensity in (C). All results have been tested at least three times. Data presented as mean ± SEM. Student's *t* test for 2-group comparisons.
